# L1CAM Expression is Related to Non-Endometrioid Histology, and Prognostic for Poor Outcome in Endometrioid Endometrial Carcinoma

**DOI:** 10.1007/s12253-016-0047-8

**Published:** 2016-02-18

**Authors:** Yvette P. Geels, Johanna M. A. Pijnenborg, Bart B. M. Gordon, Mina Fogel, Peter Altevogt, Rina Masadah, Johan Bulten, Léon C. van Kempen, Leon F. A. G. Massuger

**Affiliations:** 1 Department of Obstetrics and Gynaecology, Radboud University Nijmegen, Medical Centre, Nijmegen, The Netherlands; 2 Department of Obstetrics and Gynaecology, Elisabeth-TweeSteden Hospital, P.O. Box 90107, 5000 LA, Tilburg, The Netherlands; 3 Department of Pathology, Kaplan Medical Centre, Rehovot, Israel; 4 Tumour Immunology Program, D015, German Cancer Research Centre, 69120 Heidelberg, Germany; 5 Department of Pathology, Hasanuddin University Hospital, Makassar, Indonesia; 6 Department of Pathology, Radboud University Nijmegen, Medical Centre, Nijmegen, The Netherlands; 7 Department of Pathology, McGill University, Montreal, QC Canada; 8 Jewish General Hospital, Montreal, QC Canada

**Keywords:** Endometrial carcinoma, Immunohistochemistry, L1CAM, Histopathological diagnosis, Prognostic value, Non-endometrioid

## Abstract

The majority of endometrial carcinomas are classified as Type I endometrioid endometrial carcinomas (EECs) and have a good prognosis. Type II non-endometrioid endometrial carcinomas (NEECs) have a significant worse outcome. Yet, 20 % of the EECs are associated with an unexplained poor outcome. The aim of this study was to determine if L1CAM expression, a recently reported biomarker for aggressive tumor behavior in endometrial carcinoma, was associated with clinicopathological features of EECs. A total of 103 patients diagnosed as EEC at the Radboud University Medical Centre, based on the pathology report were selected. L1CAM status of these tumors was determined, and histologic slides were reviewed by two expert pathologists. L1CAM-positivity was observed in 17 % (18/103). Review of the diagnostic slides revealed that 11 out of these 18 L1CAM-positive tumors (61 %) contained a serous- or mixed carcinoma component that was not initially mentioned in the pathology report. L1CAM-expression was associated with advanced age, poor tumor grade, and lymphovascular space invasion. A worse five year progression free survival rate was observed for patients with L1CAM-positive tumors (55.6 % for the L1CAM-positive group, compared to 83.3 % for the L1CAM-negative group *P* = 0.01). L1CAM expression carries prognostic value for histologically classified EEC and supports the identification of tumors with a NEEC component.

**Electronic supplementary material:**

The online version of this article (doi:10.1007/s12253-016-0047-8) contains supplementary material, which is available to authorized users.

## Introduction

Endometrial carcinoma is the most common gynecological malignancy in industrialized nations [1]. The majority of endometrial carcinomas is classified as type I endometrioid endometrial carcinoma (EEC) and has a good prognosis in general. Type II carcinoma represents non-endometrioid endometrial carcinoma (NEEC), and carries a high risk of disease progression. Type I carcinomas are characterized as diploid tumors, with estrogen-, and progesterone receptors, *PTEN* alterations, microsatellite instability, mutations of *K-RAS*, and *CTNNB1*. Type II carcinomas on the contrary, are often aneuploid, and show over expression of P53 and Her2/neu [2, 3]. Yet, about 20 % of the individual cases does not fit within this dualistic model: EECs with poor clinical outcome [3, 4]. This group of endometrial carcinomas are either misclassified based on their histological appearance, or are inherently different despite truly morphological and clinical characteristics of EEC.

Recently, expression of L1 cell adhesion molecule (L1CAM) has been associated with aggressive subtypes of endometrial carcinoma [5]. Moreover, L1CAM has shown to be of great importance for the prediction of clinical outcome in FIGO-stage I, histologically confirmed EECs [6]. L1CAM is a member of the immunoglobulin super family, and a neural cell recognition molecule, implicated in embryonic brain development [7]. L1CAM has an important role in the regulation of cell-cell interactions in neurohistogenesis, including axon outgrowth, neuronal migration, and regeneration after trauma [6]. In carcinoma cell lines, L1CAM-expression augments cell motility and tumor growth.

The current study was conducted in order to identify the clinicopathological features of L1CAM-positive EECs, and to confirm prognostic value of L1CAM in EEC patients.

## Materials & Methods

### Patients and Tissue Specimen

The nationwide network and registry of histo- and cytopathology in the Netherlands (Pathologisch Anatomisch Landelijk Geautomatiseerd Archief: PALGA) was used to search for patients diagnosed, and surgically treated with hysterectomy and bilateral oophorectomy at the Radboud University Medical Centre Nijmegen for EEC’s. The terms “uterus” and “endometrioid carcinoma” were used to search through the PALGA database. Clinical data were collected by studying the medical charts. Age, menopausal state, body mass index (BMI), parity, use of estrogen, treatment, stage of disease, date of recurrence of disease, date of death, and the cause of death were registered. In case of missing values the case was not used for the specific calculations. Stage of disease was based on the 2009 International Federation of Gynecology and Obstetrics (FIGO) staging system. Four to eight representative slides of all patients were retrieved from the pathology archive and used for review. Review was done systematically including the following items: tumor grade, depth of myometrial invasion, the presence of lymphovascular space invasion, and the histological type. Review was performed independently by an experienced pathologist (RM) and an expert gyneco-pathologist (JB), who were unaware of the original pathology report, and the clinical outcome of the patient. Initial diagnosis was compared with the diagnosis after review. In case of discrepancy, the final diagnosis was obtained by consensus between the two pathologists. Confirmed EECs were also included in the study of Zeimet et al. [6] Immunohistochemical analysis of L1CAM was performed on sections of all endometrial carcinomas. The stained sections were analyzed by an independent pathologist who was not aware of the clinical outcome of the patients.

### Antibodies and Immunohistochemistry

A monoclonal antibody to L1CAM (L1-40.10) was obtained after immunization of mice with human L1-Fc protein comprising the ectodomain of L1. Staining was performed as described previously [6]. Briefly, following EDTA antigen retrieval, sections were stained using the automated I6000 immunostainer (Biogenics, San Ramos, California, USA), staining of tissue was visualized using 3,3′-Diaminobenzidine (Zymed lab. California, USA) as substrate, and counterstained with Mayer’s haematoxylin. Positive staining was defined as >10 % immunoreactivity in any section derived from the tumor. Strong L1CAM expression in nerve bundles of deeper accompanying connective tissues was used as internal positive control.

### Statistical Analysis

Differences in clinicopathological parameters between the group of patients with L1CAM-positive and L1CAM-negative tumors were tested for statistical significance using the Pearson’s Chi-Square (χ^2^) test, or the Fisher’s exact test, and Mann-Whitney test. All *P*-values presented are two-sided, and associations were considered significant if the *P*-value was less than 0.05. Survival analyses were performed to study the progression free survival (PFS). PFS was calculated from the date of surgery until the last date of progression free follow-up. The prognostic impact of clinicopathological parameters were analyzed by using univariable and multivariable Cox proportional hazards models. The forward stepwise method was used for selection procedures. These results were expressed as hazard ratio’s (HR) with their 95 % confidence intervals (95 % CI). All statistical analyses were performed using the software package SPSS 20.0 (SPSS Inc).

## Results

### Patient Characteristics and Treatment

A total of 103 patients with EEC were selected for this study, and included for analysis. Patient characteristics are presented in Table 1. Lymph node dissection was performed only in patients suspected of advanced stage and/non-endometrioid histology according the Dutch guideline for endometrial cancer treatment.

**Table 1:** Clinical and pathologic characteristics (after review) in the total population (*n* = 103)

Clinico-pathologic characteristics	Total (*n* = 103)
Median age in years (range)	63 (24–86)
Postmenopausal	
No	22 (21.3 %)
Yes	73 (70.9 %)
Unknown	8 (7.8 %)
Median BMI* in kg/m^2^ (range)	28.9 (18.7–53.6)
Lymph nodes	
Positive	1 (1.0 %)
Negative	22 (21.3 %)
Unknown	80 (77.7 %)
Adjuvant radiotherapy	
Yes	39 (37.9 %)
No	64 (62.1 %)
Adjuvant chemotherapy	
Yes	1 (1.0 %)
No	102 (99.0 %)
FIGO stage**	
Low (I-II)	84 (81.6 %)
High (III-IV)	19 (18.4 %)
Tumor grade	
Low (1–2)	78 (75.7 %)
High (3)	25 (24.3 %)
Myometrial Invasion	
< 50 %	61 (59.2 %)
> 50 %	42 (40.8 %)
Lymphovascular Space Invasion	
Not present	80 (77.7 %)
Present	23 (22.3 %)
Histology	
Endometrioid	92 (82.3 %)
Non- endometrioid	11 (10.7 %)
Adjuvant radiotherapy	
No	64 (62.1 %)
Yes	39 (37.9 %)
Adjuvant chemotherapy	
No	100 (99.0 %)
Yes	1 (1.0 %)
Five year disease specific survival rate	88.8 %
Five year progression free survival rate	77.7 %
Median follow up (months) (range)	57 (0–148)

*Body Mass Index

**1988 International Federation of Gynecology and Obstetrics staging system

### Review of the Histological Slides

After review, the initial diagnosis was adjusted in 31 patients. In 25 cases tumor grade changed. In 11 cases the histology was classified different. Five tumors were finally diagnosed as uterine papillary serous carcinoma (UPSC), three tumors were diagnosed as mixed carcinoma, and three as undifferentiated carcinomas. The diagnosis of mixed carcinoma was defined when at least 10 % of a second component was present. The differences in initial diagnosis, and diagnosis after review are shown in Supplemental Digital Content Table 1.

### Immunohistochemistry

**Fig. 1:**
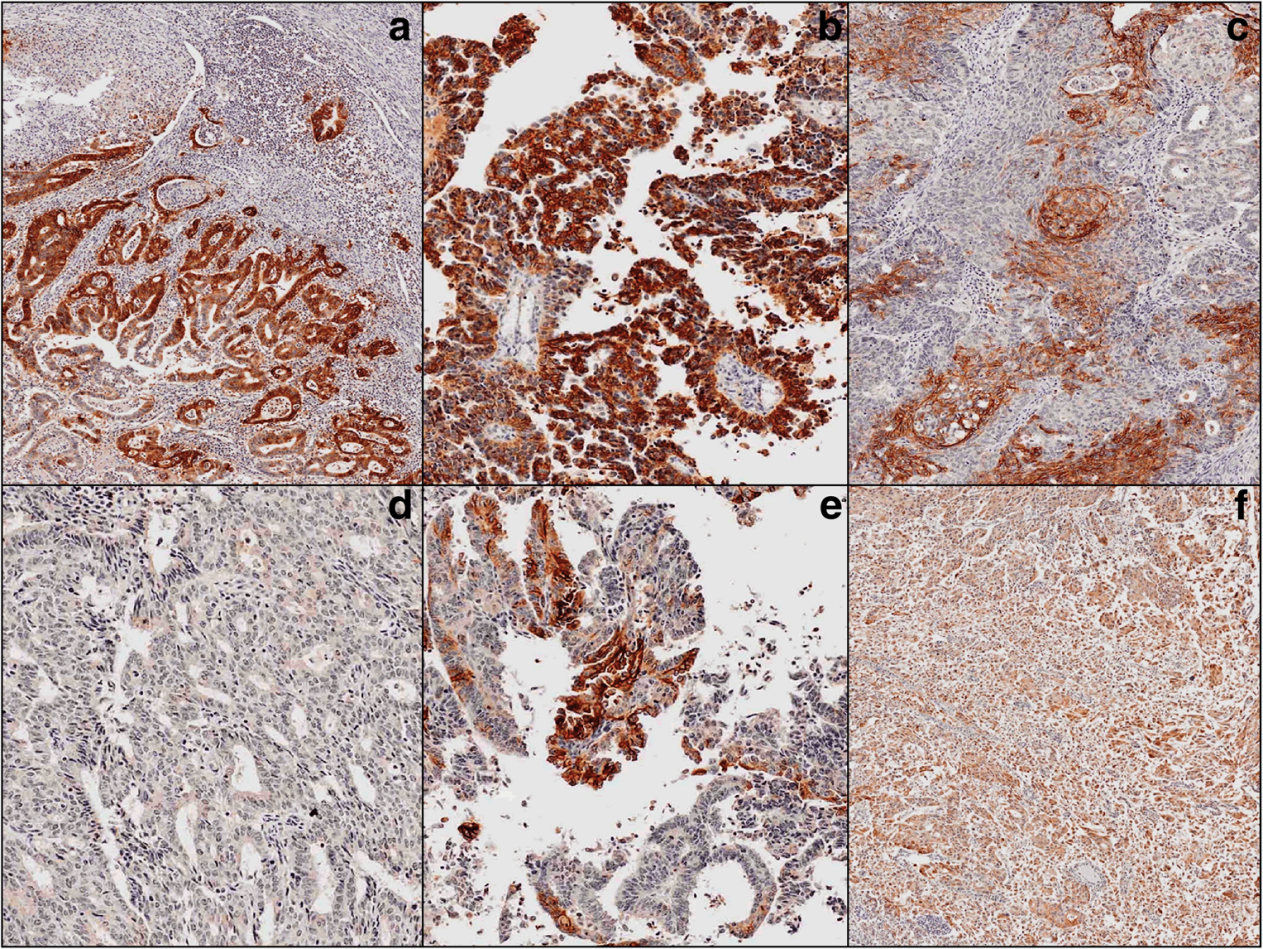
Endometrioid endometrial carcinoma with L1-CAM positive staining at the invasive front (**a**), Papillary serous carcinoma with L1-CAM positive staining (**b**), Endometrioid carcinoma with L1-CAM positive staining (**c**), Mixed carcinoma with 50 % endometrioid component and 50 % serous component, the endometrioid component with L1-CAM negative staining (**d**), Mixed carcinoma with 50 % endometrioid component and 50 % serous component, the serous component with L1-CAM positive staining (**e**), Undifferentiated carcinoma with L1-CAM positive staining (**f**)

L1CAM is positivity was particularly present, the cell membranes of the tumor cells, and only weakly present in the cytoplasm of these positive tumor cells. Neither stromal cells nor inflammatory cells were stained by L1CAM. In the study population of 103 carcinomas, 18 patients showed L1CAM-positive staining in the tumor (17 %). All cases (*n* = 11) that were classified as NEEC after revision were L1CAM positive (100 %), whereas only seven cases of confirmed EECs were L1CAM-positive (7,6 %). L1CAM staining showed variable intensity. Staining had a tendency to more intensity at the invasive front (Fig. 1a). The five tumors, diagnosed as pure UPSC after review of the histological slides, showed L1CAM-expression throughout a major part of the tumor specimens. A representative example is shown in Fig. 1b. Figure 1c shows an example of L1CAM-expression in EEC. In the two mixed carcinomas with 50 % serous component, and 50 % endometrioid component, the serous component was strongly positive, whereas the endometrioid component was weakly positive, or L1CAM-negative (Fig. 1d and e). Figure 1f shows L1CAM-expression in an undifferentiated carcinoma, which is diffuse positive trough the tumor specimen.

### L1CAM Expression and Clinicopathological Characteristics

The clinicopathological characteristics of L1CAM-negative and L1CAM-positive patients after review are summarized in Table 2. Adjuvant treatment and time of follow up were equal in both groups. Patients with L1CAM-negative tumors were significantly younger compared to patients with L1CAM-positive tumors. In addition, L1CAM-expression was associated with, poor tumor grade (HR 3.80 (1.61–9.00)), and lymphovascular space invasion (HR 4.13 (1.73–9.86)). A worse five year progression free survival rate was observed for patients with L1CAM-positive tumors (55.6 % for the L1CAM-positive group, compared to 83.3 % for the L1CAM-negative group *P* = 0.01).

**Table 2:** Clinical and pathologic characteristics (after review) in the total population, the L1CAM-negative- and the L1CAM-positive tumours

Clinico-pathologic characteristics	L1CAM-negative *n* = 85	L1CAM-positive *n* = 18	*P*-value
Mean age in years (range)	59.7 (24–86)	68.2 (47–81)	<0.01
Postmenopausal			
No	21 (26.3 %)	1 (6.7 %)	
Yes	59 (73.7 %)	14 (93.3 %)	0.18
Median BMI in kg/m^2^ (range)	29.3 (18.7–53.6)	27.1 (19.8–47.1)	0.12
Lymph nodes			
Positive	1 (5.6 %)	0 (27.8 %)	
Negative	17 (94.4 %)	5 (100.0 %)	1.00
FIGO stage*			
Low (I)	66 (77.6 %)	12 (66.7 %)	
High (II-IV)	19 (22.4 %)	6 (33.3 %)	0.37
Tumor grade			
Low (1–2)	75 (88.2 %)	3 (16.7 %)	
High (3)	10 (11.8 %)	15 (83.3 %)	<0.01
Myometrial Invasion			
< 50 %	54 (63.5 %)	7 (38.9 %)	
> 50 %	31 (36.5 %)	11 (61.1 %)	0.06
Lymphovascular Space Invasion			
Not present	73 (85.9 %)	7 (38.9 %)	
Present	12 (14.1 %)	11 (61.1 %)	<0.01
Histology			
Endometrioid	85 (100 %)	7 (38.9 %)	
Non- endometrioid	0 (0.0 %)	11 (61.1 %)	<0.01
Radiotherapy			
No	49 (57.6 %)	13 (81.3 %)	
Yes	36 (42.4 %)	3 (18.8 %)	0.10
Mean follow-up in months (range)	60.4 (0.4–148.0)	51.1 (0–147.0)	0.40

*2009 International Federation of Gynecology and Obstetrics staging system

## Discussion

This study was conducted to determine whether L1CAM-expression in EECs is related to pathological features and clinical outcome. Our study revealed L1CAM-expression in 18/103 (17 %) of the tumors originally diagnosed as EEC. Eleven of these cases were reclassified as NEEC after expert review of the original diagnostic slides, and seven L1CAM-positive EECs were identified. These data illustrate that L1CAM expression can be supportive for the identification of NEEC.

The study group of 103 endometrial carcinoma patients is representative for the population diagnosed with endometrial cancer, with a median age at diagnosis of 63 years, a median BMI of 28.9 kg/m^2^, a majority of early stage disease, and a minority of poorly differentiated tumors. Yet, the study is limited by its relative low number of cases, and the retrospective character of the study.

A recent multicenter study of L1CAM-expression in 1021 histologically confirmed EECs demonstrated L1CAM positivity in 17,7 % and demonstrated that L1CAM-expression in EEC was an independent predictor of clinical outcome. A small percentage of these cases showed areas of non-endometrioid differentiation in less than 10 % of the tumor, and this was associated with L1CAM-expression [6]. Interestingly, in the current study an equal percentage of L1CAM positivity was initially observed, yet after revision and reclassification L1CAM expression in EEC was observed in 7,6 %. Our findings are in line with data of Bosse et al. who observed a significantly lower percentage of L1CAM positivity in EEC [8]. The current study confirms that L1CAM-expression carries prognostic value for histologically classified EEC, but also supports the identification of tumors with a NEEC component. The recognition of UPSC can be challenging, and it is expected that improved awareness of the pathologist of the existence of UPSC nowadays, will decrease the number of UPSCs mistaken for EECs.

**Fig. 2:**
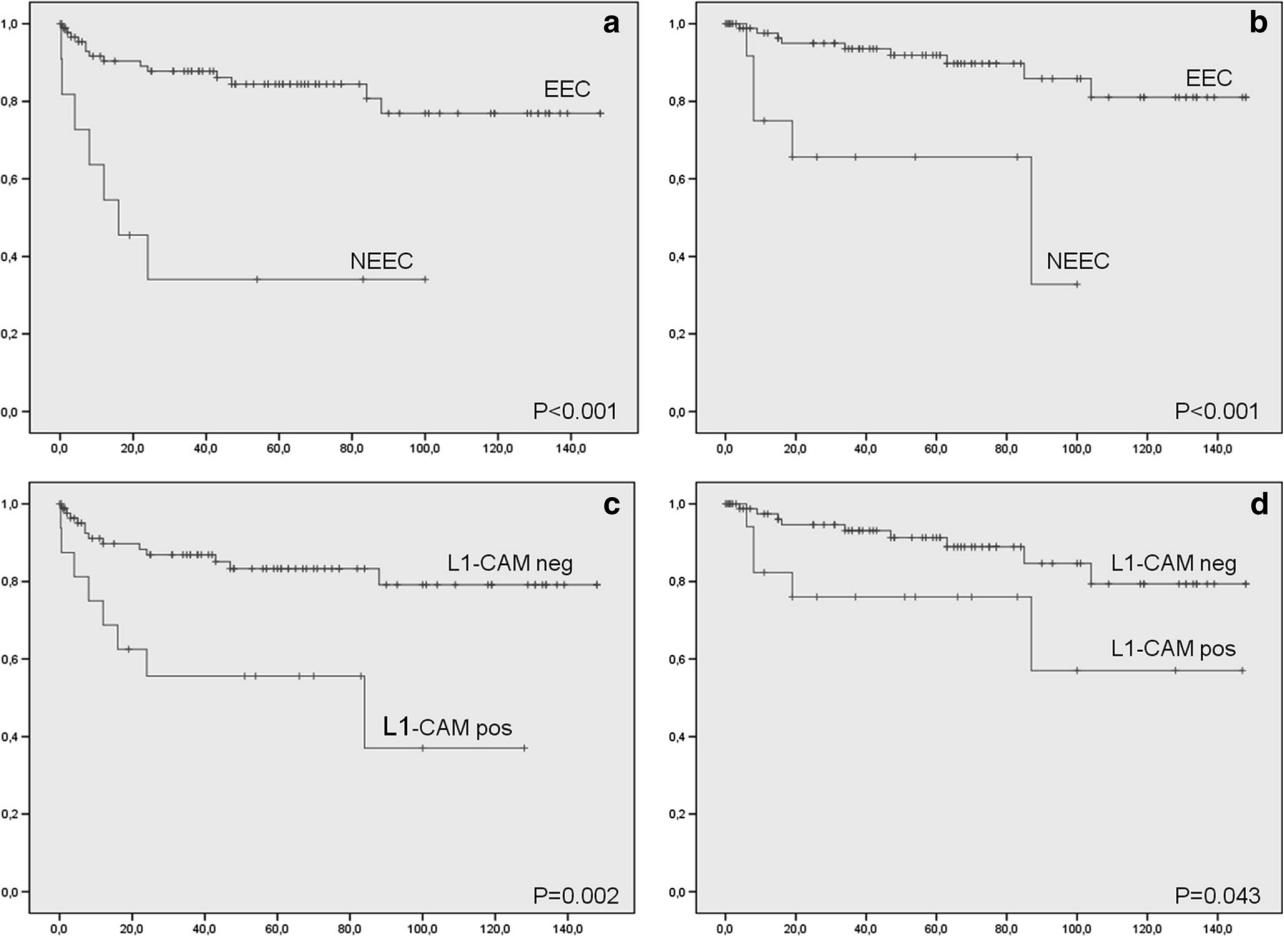
Progression free survival in EEC patients compared with NEEC patients in months (**a**), Disease specific survival in EEC patients compared with NEEC patients in months (**b**), Progression free survival in L1-CAM negative patients compared with L1-CAM positive patients in months (**c**), Disease specific survival in L1-CAM negative patients compared with L1-CAM positive patients in months (**d**)

Several studies have shown that L1CAM-expression is associated with aggressive carcinoma subtypes and tumor progression [9]. In serous ovarian and endometrial carcinomas, L1CAM-expression is frequently present [9]. If L1CAM-expression is present in EECs, it is associated with poor tumor differentiation, absence of estrogen- and progesterone receptors, and loss of E-cadherin expression [5]. In our study, L1CAM-expression was associated with predictors of poor clinical outcome like tumor grade and LVSI. As a consequence, L1CAM expression was associated with a significantly decreased 5-year progressive free survival. L1CAM expression was associated with recurrent disease, but not specific to distant metastasis like previously published, but due to the small numbers these results should be interpreted with caution Fig. 2.

Currently, risk estimation in endometrial cancer is based on both preoperative and postoperative factors. Data of the MoMaTEC trial demonstrated that loss of hormone receptor status in the preoperative tumor specimen was significantly associated with lymph node metastasis [10].

In this pre-operative setting L1CAM could be a useful additional tool. It not only helps to identify NEECs, but it also identifies those EEC-patients who are at high risk of disease progression.

Determination of histology in poorly differentiated tumors can be challenging. Yet, accurate diagnosis of endometrial carcinomas is of great clinical importance. In this study, the mixed endometrial carcinomas showed L1CAM-expression in the serous component, whereas the endometrioid component was L1CAM-negative. These observations have been described previously [5]. In literature, several immunohistochemical markers are used to support the diagnosis of UPSC, i.e. over-expression of p53, and p16, as well as loss of hormone receptors. The current study demonstrates that L1CAM might be a useful marker to distinguish EEC from NEEC. In addition, L1CAM-staining was observed to be strongest at the invasive front, which confirms the suggestion that L1CAM is important for tumor invasion. However in the to date largest reported study on L1CAM-expression in EEC, this particular pattern of L1CAM-staining was not observed [6].

In conclusion, L1CAM is significantly associated with non-endometrioid histology and other clinicopathological factors predicting poor survival. This makes L1CAM a potential marker for pre-operative identification of patients needing aggressive treatment. Furthermore, L1CAM could be a useful marker in the detection of non-endometrioid histology and of EEC with poor prognosis. A large prospective study is required to determine the clinical implications of L1CAM in endometrial carcinomas.

## Electronic supplementary material

Supplemental Table 1 (DOCX 14 kb)

Supplemental Table 2 (DOCX 15 kb)
