# Supplementary material for: L1CAM Expression is Related to Non-Endometrioid Histology, and Prognostic for Poor Outcome in Endometrioid Endometrial Carcinoma
Source: Pathol Oncol Res. 2016 Feb 18;22(4):863–8. doi: 10.1007/s12253-016-0047-8 (PMC5031726; doi:10.1007/s12253-016-0047-8)
Supplement: Supplementary file 1 — (DOCX 14 kb) [file 12253_2016_47_MOESM1_ESM.docx]

**Supplemental Digital Content 1: Initial diagnosis and diagnosis after review of cases with a changed diagnosis of EEC into NEEC**

|  | Before revision | | After revision | |
| --- | --- | --- | --- | --- |
| Case | Histology | Grade | Histology | Grade |
| 6 | Endometrioid | 3 | Serous | 3 |
| 12 | Endometrioid | 3 | Serous | 3 |
| 14 | Endometrioid | 2 | Undifferentiated | 3 |
| 18 | Endometrioid | 2 | Serous | 3 |
| 21 | Endometrioid | 2 | 50% endometrioid / 50% serous | 3 |
| 32 | Endometrioid | 3 | Serous | 3 |
| 45 | Endometrioid | 2 | 50% endometrioid / 50% serous | 3 |
| 47 | Endometrioid | 3 | Undifferentiated | 3 |
| 48 | Endometrioid | 3 | Serous | 3 |
| 50 | Endometrioid | 2 | 50% serous/ 50% clear cell | 3 |
| 53 | Endometrioid | 3 | Undifferentiated | 3 |

*­­­­*
