# Supplementary material for: L1CAM Expression is Related to Non-Endometrioid Histology, and Prognostic for Poor Outcome in Endometrioid Endometrial Carcinoma
Source: Pathol Oncol Res. 2016 Feb 18;22(4):863–8. doi: 10.1007/s12253-016-0047-8 (PMC5031726; doi:10.1007/s12253-016-0047-8)
Supplement: Supplementary file 2 — (DOCX 15 kb) [file 12253_2016_47_MOESM2_ESM.docx]

**Supplemental Digital Content 2 only EEC patients**

| Clinico-pathologic characteristics | L1CAM-negative  n=85 | L1CAM-positive  n=7 | P-value |
| --- | --- | --- | --- |
| Mean age in years (range) | 59.7 (24-86) | 64.3 (47-)78 | 0.26 |
| Postmenopausal  No  Yes | 21 (26.3%)  59 (73.7%) | 1 (14.3%)  6 (85.7%) | 0.67 |
| Median BMI in kg/m^2^ (range) | 29.3 (18.7-53.6) | 27.1 (19.8-47.1) | 0.23 |
| Lymph nodes  Positive  Negative  FIGO stage*  Low (I)  High (II-IV) | 1 (5.6%)  17 (94.4%)  66 (77.6%)  19 (22.4%) | 5 (1000.0%)  0 (0.0%)  6 (85.7%)  1 (14.3%) | 0.924  1.00 |
| Tumor grade  Low (1-2)  High (3) | 75 (88.2%) 10 (11.8%) | 3 (42.9%) 4 (57.1%) | <0.01 |
| Myometrial Invasion  <50%  >50% | 54 (63.5%)  31 (36.5%) | 4 (57.1%)  3 (42.9%) | 0.71 |
| Lymphovascular Space Invasion  Not present  Present | 73 (85.9%)  12 (14.1%) | 2 (28.6%)  5 (71.4%) | <0.01 |
| Radiotherapy  No  Yes | 49 (57.6%)  36 (42.4%) | 7 (100.0%)  0 (0.0%) | 0.04 |
| Mean follow-up in months (range) | 60.4 (0.4-148.0) | 83.7 (37.0-147.0) | 0.16 |

*1988 International Federation of Gynecology and Obstetrics staging system
